# Notes and News

**Published:** 1890-06-07

**Authors:** 


### NOTES AND NEWS.

COLLECTED AND COMMUNICATED.

Their Royal Highnesses the Prince and Princess of Wales have consented to open the new branch hospital of the Seamen's Hospital Society at the Royal Victoria and Albert Docks, E., on Tuesday, the 24th of June next.

The annual report of the Royal Free Hospital in Gray's Inn Road is very full and satisfactory; the committee earnestly point out the necessity for the new building, and also the need of further annual subscribers. Eighty lady medical students passed through the hospital in 1889; it is notable that ladies figure largely among the annual subscribers.

A wayfarer lately, in a primitive part of Kent, inquired of a rustic whom he met whether there was a doctor near, as he had hurt his foot and wanted it looked to. "Doctor, sir?" said the man, with a knowing shake of his head, "There ain't no such thing about here. If we sprains ourselves, or has the toothache, we goes to the blacksmith; but, thank God, we all dies natural deaths."

Before leaving the London Hospital on the 20th inst., the Duke of Cambridge wrote in the visitors' book: "I rejoice in having had the opportunity this day of laying a foundation-stone for the further extension of this great and noble hospital, which has already done so much for the sick and poor of this large neighbourhood, by which I trust its usefulness will be further increased for future generations."

The Public Sanitary Inspectors visited Leamington on May 24th, and spent a most enjoyable day. The Mayor entertained them at lunch, and the Countess of Warwick provided them with tea. Sir E. Chadwick and Sir Douglas Galton supplied the mental food, and visits to the Sewage Works, Warwick Castle, Earl Leycester's Hospital, and other places of interest, filled in a very pleasant and profitable programme.

Forthcoming events which our readers should note include a bazaar at the Athenæum, Camden Road, on June 9th, 10th, and 11th, in aid of the North-West London Hospital; the Annual Court of the Victoria Hospital for Children at Chelsea, on June 11th, at 3.45, at which Princess Louise will preside; and the opening of the new branch of the Dreadnought at the docks by the Prince and Princess of Wales on June 24th.

The following vacancies are announced this week, the amount of the salary in each case being given after the name of the institution:—

**Resident Medical Officers.**

Victoria Park Chest Hospital, House Physician—board, etc.
West London Hospital, House Physician—board, etc.
West London Hospital, House Surgeon—board, etc.
City of London Lunatic Asylum, Assistant Medical Officer—£150, board, etc.
Carmarthen Infirmary, House Surgeon—£100, board, etc.
University College, Resident Medical Officer.
Seamen's Hospital Society, House Surgeon.
Miller Hospital, Greenwich, Junior Medical Officer—£30, board, etc.
Bristol General Hospital, House Surgeon—£120, board, etc.

**Non-Resident Medical Officers.**

Parish of Farr, Sutherland, Medical Officer—£100.
London Hospital, Assistant Physician and Assistant Surgeon.

A lady writes to us to say that she was lately visiting a small child at a hospital, and took with her a large doll; but the doll did not please, and the small patient begged for a horse and cart. It was a Saturday afternoon, however, and no horse and cart could be had. Our correspondent suggests that it would be to the advantage of the hospital and of the patients to have a toy stall in the building. Alas! so much is already demanded of hospital authorities we fear they will not jump at this suggestion.

The annual display of the National Physical Recreation Society at the Agricultural Hall, Islington, opened on May 26th under very favourable auspices. The Prince of Wales, who arrived a few minutes after half-past seven p.m., received a very cordial welcome from the audience. The programme was a very varied one, and the various items were favourably received. Special mention must be made of the physical exercises by the girls in charge of Mrs. Strachan Matthews, and the musical drill by students of the Royal Normal College and Academy of Music for the Blind.

Mr. J. S. Wood, the Treasurer of the Royal South London Ophthalmic Hospital writes:—"Will you permit me to add to the note you kindly inserted to the effect that the Prince of Wales had promised to lay the foundation stone in July, of the new building for the hospital (which was established 33 years ago), that H.R.H. the Princess of Wales has now graciously agreed to receive purses of £5 5s. and upwards from ladies and children who will present them to Her Royal Highness on behalf of the Royal Foundation Fund. I shall be happy to send purses to any of your readers, and as the purses will be returned to the collectors after the ceremonial with an inscription, they will form a pleasing momento of an important event in connection with a worthy and much needed a chrity."

Once more we call attention to the Mendicity Society, to which all tender-hearted ladies should belong. The great point about this Society is that it supplies its members with food-tickets, which can be given to the pleading beggar, and will provide him with food if he really needs it, but will not give him the drink he probably craves. In moving the adoption of the report lately, Baron F. de Rothschild observed that four millions of money were spent every year on hospitals, while another three millions were given to the needy, but not always to the deserving. The Society, through its plain-clothes officers, discovered and sometimes prosecuted impostors, and distributed relief to deserving cases. While moving "That this Society begs humbly to express its deep sense of Her Majesty's favour in continuing her Royal patronage and support to the institution," Sir William Fraser first referred to the manner in which charity was frequently misplaced, and then to the method adopted by the Mendicity Society, by which every shilling was properly placed. Over a thousand begging-letters were investigated last year, and over 7,000 food tickets given away.

